# Chemical-nutritional parameters and volatile profile of eggs and cakes made with eggs from ISA Warren laying hens fed with a dietary supplementation of extruded linseed

**DOI:** 10.5713/ajas.19.0309

**Published:** 2019-08-26

**Authors:** Andrea Ianni, Fiorentina Palazzo, Lisa Grotta, Denise Innosa, Camillo Martino, Francesca Bennato, Giuseppe Martino

**Affiliations:** 1 Faculty of BioScience and Technology for Food, Agriculture and Environment, University of Teramo, Teramo 64100, Italy; 2 Istituto Zooprofilattico Sperimentale dell'Abruzzo e del Molise “G. Caporale”, Via Campo Boario 37, 64100 Teramo, Italy

**Keywords:** Linseed, Laying Hen, Egg Yolk, Volatile Compounds, Linolenic Acid, Cholesterol

## Abstract

**Objective:**

The aim of this study was to evaluate the chemical-nutritional parameters, oxidative stability and volatile profile of eggs and cakes made with eggs from laying hens fed with a dietary supplementation of extruded linseed.

**Methods:**

Two thousand ISA Warren laying hens were randomly divided into two groups: a control group was fed with a standard diet while the experimental group received the same diet supplemented with 7% of extruded linseed. The trial lasted 84 days, in which three samplings of laid eggs were performed. Samples of eggs and food systems arising from eggs were then analyzed in order to obtain information about β-carotene and total flavonoid content, antioxidant activity, fatty acid profile, lipid oxidation, and volatile profile.

**Results:**

Linseed induced the increase of α-linolenic acid with consequent reduction of the ω-6/ω-3 ratio (4.3:1 in egg yolk); in addition to this, was evidenced the cholesterol reduction and the significant increase in total flavonoids and β-carotene, although no variations were detected in antioxidant capacity. Even in cooked products there was not only a direct effect of linseed in increasing α-linolenic acid, but also in inducing the reduction of cholesterol and its major oxidation product, 7-ketocholesterol. The dietary linseed integration was also shown to affect the volatile profile of baked products.

**Conclusion:**

Data confirmed that dietary supplementation with extruded linseed resulted in food products with interesting implications for human health. With regard to the volatile profile of baked products it would be necessary undertake further sensorial analysis in order to evaluate any variations on flavor and consumer acceptability.

## INTRODUCTION

Hen egg represents a traditional food with an excellent nutritive value due to the presence of highly digestible proteins, vitamins, minerals and lipids, such as phospholipids and polyunsaturated fatty acids (PUFA) [1]. Commercial eggs are characterized by high proportions of dietary cholesterol and ω-6 PUFA, and low concentration of ω-3 fatty acids (FAs). Lipid composition of hen’s eggs is a subject of primary consumer concern, due to the relationship between specific dietary lipids and the development of conditions which heavily influence human health and life expectancy, for example coronary heart diseases (CHD) and atherosclerosis [2]. It is generally accepted that ω-3 PUFAs (n-3 PUFA) provide important health benefits to humans [3], which also involved prevention and treatment of many chronic diseases, such as neurological disorders, cancer, inflammatory diseases, obesity and diabetes mellitus [4]. The most representative n-3 PUFAs are α-linolenic acid (18:3 n-3; ALA) eicosapentaenoic acid (20:5 n-3; EPA) and docosahexaenoic acid (22:6 n-3; DHA). Initially, all studies highlighted the concept that greatest benefits to human health were mostly associated to the role of EPA and DHA, while ALA, an essential FA, was credited to play a role only as a substrate for the synthesis of EPA and DHA [5]. Later, an increasing number of epidemiological and controlled studies, have demonstrated the ability of ALA obtained from plants to reduce the risk of CHD [6–8].

For the mentioned reasons, over the course of time a growing interest has developed in the production of eggs rich in ω-3 FAs, by feeding laying hens with experimental feedstuffs containing these nutrients because of the close correlation between their levels in diet and in the yolk [9]. Eggs enriched with ω-3 FAs were obtained by including oil seeds or oils from terrestrial and marine sources in poultry diets. The ALA is predominantly located in the chloroplast of green leafy vegetables and also in seed oils such as flax (*Linum usitatissimum*), canola (*Brassica napus*), chia (*Salvia hispanica*), and camelina sativa (*B. napus*) [10].

Flaxseed is one of the most important oilseed crops for industrial as well for food and feed purposes; every part of the linseed plant is utilized commercially, either directly or after processing. The stem yields good quality fiber having high strength and durability. The seed provides oil rich in omega-3, digestible proteins, and lignans. Flaxseed contains a seed coat or true hull (also called testa), a thin endosperm, two embryos, and an embryo axis. Embryos form 55% of the total weight of hand-dissected flaxseed, the seed coat and the endosperm account for 36% of the total weight, and the embryo axis is 4%. Flaxseed is unique among oilseeds because of its exceptionally high content of ALA and lignans; flaxseed contains 35% to 45% oil, of which 45% to 52% is ALA [11,12].

Animal food products such as eggs are very resistant to oxidative reactions due to presence of natural antioxidants, including vitamin E, avidin, and phosphatine. However, the long chain ω-3 FAs possess a characteristic “fishy” or “paint-like” aroma, so there is concern that alteration of ω-3 FA content may affect the eating (sensory) quality of the food products [4,10]. The objective of this research was to determine the effect of extruded linseed (EL) supplemented diets on hen’s performance, FAs composition, oxidative stability and volatile profile of resultant eggs and food systems arising from eggs.

## MATERIALS AND METHODS

### Experimental design, diets, and sampling

All procedures related to animals’ care, handling and sampling were conducted in compliance with current legislation on animal welfare (D.Lgs. 267/2003 of the Italian Parliament). For the scope of the study, animals did not undergo breeding practices other than those commonly adopted, for this reason it is not considered necessary to provide further ethical declarations. Two thousand ISA Warren laying hens with an age of 20 weeks were used in this study. Hens were randomly divided into two groups of 1,000 each: a control group (CG) and an experimental group (EG) whose diet was supplemented with EL. The study was conducted for a period of 84 days (after 7 days from restocking), in which animals of each EG were housed in two separate areas with an ambient temperature of 20°C±2°C and a photoperiod of 16 h of light and 8 h of darkness. Water was provided *ad libitum*, through common water-trough. All animals received an isoproteic and isoenergetic diet for laying hens in production whose chemical composition is reported in Table 1; the diets only differed for the 7% EL supplementation administered to EG. This parameter was defined taking into account the information previously reported in similar studies, in which variations of eggs chemical composition were observed for a degree of supplementation rarely in excess of 10% [3]. Weekly from the beginning of the trial, the food and water intake in both the EGs was assessed and the percentage of deposition was determined.

The laid eggs were collected at the 28th, 56th and 84th day from the beginning of the trial. Each sampling involved the collection, from each EG, of 200 eggs which were first used to determine the physical parameters (egg albumen, yolk and shell weights, but also the yolk color) and then chemical analyzes were performed on both fresh yolks and baked products (cakes). With regard to cakes, at each sampling a dough was prepared for each EG, with the following composition: 200 g of yolk obtained from CG and EG respectively, 240 g of sugar, 240 g of flour, 60 g of water and 20 g of yeast. After homogenization, the mixtures were divided into 6 cm diameter cake molds. Specifically, 15 cakes were prepared for each EG. Three distinct firings were performed in each of which were cooked 10 cakes, 5 prepared with egg yolks obtained from the CG and 5 with egg yolks from the EG. In the three firings, cakes were cooked with three different timings, 15, 30, and 45 minutes, reaching in the cake cores the temperature of 110°C, 149°C, and 150°C, respectively. During the cooking procedures, the oven temperature was kept constant at 180°C± 5°C. The oven temperature and the core temperature of the cakes were recorded every 5 minutes through the use of a thermocouple (FASINT’, Milan, Italy). At the end of cooking, cakes were placed immediately at −20°C in order to instantly block any oxidation process and stored until analysis. The fresh yolks and the related processed products not immediately used after sampling were vacuum-packed and stored at −20°C until further analysis.

### Evaluation of egg yolks color and determination of β-carotene

Egg yolk color was evaluated by using the color measuring device Konica Minolta Chroma Meter CR-300 (Minolta corp., Ramsey, NJ, USA). In order to perform the evaluation, samples of egg yolk were located in a glass cell with an optically clear bottom; a white standard tile was used to calibrate the chroma meter. The parameters have been determined according to the Commission Internationale de L’Eclairage (CIE) L*a*b* system, in which L* defines the color space in terms of lightness (low values owards black and higher values towars white), a* represents the redness (negative towards green, positive towards red) and b* is associated to yellowness (negative towards blue, positive towards yellow). L* is expressed in percentage (0 for black and 100 for white) whereas a* and b* are represented by values ranging from −60 to +60. For each measurement, the instrument was set perpendicular to the egg yolk surface.

Rapid determination of β-carotene was performed by the AOAC method 43.015 [13]. One gram of egg yolk was weighed, mixed with 500 μL of milli-Q water and placed in a 50 mL beaker in which were added 10 mL of acetone. Everything was shaken and placed in the dark for 30 minutes at room temperature. The solution was then filtered and further acetone was added until the final volume of 20 mL was reached. Two mL of the solution were used for the spectrophotometric reading at 450 nm. In order to dose the pigment, was prepared a linear calibration curve with different concentrations of β-carotene (Sigma-Aldrich, Milan, Italy) in the range 1 to 100 μg/mL (R^2^ = 0.9965).

### Determination of total flavonoids and antioxidant activity in extruded linseed, feeds, eggs and cakes

The phenolic compounds were extracted from EL and feeds according to the procedure previously reported by Tsakona et al [14]. Extraction was performed in a 70% (v/v) ethanolic solution that was left in the dark at 45°C for 8 hours and subsequently filtered [14].

The total flavonoid content was determined by the spectrophotometric method of aluminum chloride [15]. Five hundred μL of extract were mixed with 2.5 mL of ethanol and 500 μL of a 2% methanolic solution of aluminum chloride hexahydrate and the resulting solution was left at room temperature in the dark for an hour. Then it was possible to proceed with the spectrophotometric evaluation at 420 nm. For the quantification a calibration curve was created with quercetin (R^2^ = 0.9876) and the results were expressed as μg of quercetin equivalents/mL (μg QUE/mL).

Evaluation of antioxidant activity (AA) was performed by using the 2,2’-azinobis-(3-ethylbenzothiazoline-6-sulfonic acid (ABTS) method according to the procedure of Chen et al [16] with slight modifications. The ABTS+ radical was diluted until an absorbance of 0.7±0.2 was reached at a wavelength of 730 nm. In each cuvette 1 mL of appropriately diluted ABTS and 100 μL of each sample were added to start the reaction. Absorbance was recorded after one minute and up to 6 minutes from the sample addition. An external calibration curve with Trolox (R^2^ = 0.9949) was prepared for quantification of the antioxidant activity and the results were expressed as Trolox equivalent antioxidant capacity in μmol/mL [16].

### Analysis of fatty acids profile in extruded linseed, feeds, eggs and cakes

In all the analyzed matrices the lipid extraction was performed by using a mix of chloroform and methanol (2:1, v/v). Trans-methylation of lipid extracts and separation of fatty acyl methyl esters (FAMEs) were performed following the procedure reported by Ianni et al [17] with slight modifications. For each analyzed sample, 50 mg of lipids were reconstituted with 1 mL of hexane containing C21:0 methyl ester as internal standard (Sigma-Aldrich, Italy), and 500 μL of sodium hydroxide in methanol were used for methylation. Separation of FAME was performed by using a gas chromatograph (Focus GC; Thermo Scientific, Waltham, MA, USA) equipped with a capillary column (CP88-select CB for FAME; 0.25 μm film thickness, 100 m×0.25 mm i.d.; Agilent Technologies, Santa Clara, CA, USA) and a flame ionization detector (FID). Hydrogen was used as a carrier gas. The initial holding temperature was 175°C for 1 min, then it was increased to the final temperature of 215°C at a rate of 2°C/min and kept constant for 20 min. Peak areas were quantified using ChromeCard software, and the relative value of each individual FA was expressed as a percentage of the total FA. The value of each FA was used to calculate the sum of saturated fatty acid (SFA), monounsaturated fatty acid (MUFA), and PUFA.

### Evaluation of oxidative stability by thiobarbituric acid reactive substances-test

Lipid oxidation in egg yolks and cakes was evaluated by measuring thiobarbituric acid reactive substances (TBARs). The analysis was performed according to the procedure reported by Grotta et al [18] with slight modifications. For each sample, an aliquot of 3 g of frozen sample was mixed, within 2 min of sample withdrawal from the freezer, with 300 μL of 0.1% of butylated hydroxytoluene (BHT) in methanol to stop the oxidation process. The mixture was homogenized with Ultra Turrax T-25 high speed homogenizer (IKA, Staufen, Germany) in 30 mL of an acqueous solution of 7% trichloroacetic acid, and then subjected to distillation. An aliquot of 2 mL of each distillate was mixed with an equal volume of a 0.02 M thiobarbituric acid solution in 90% acetic acid. The solution was kept for one hour in a thermostated bath at 80°C, and only after cooling was the absorbance evaluated with a spectrophotometer (Jenway, Essex, UK); the wavelength was set at 534 nm. The amount of oxidized compounds of each sample was calculated by using a calibration curve and results were expressed in μg of malondialdehyde (MDA) per g of fresh sample.

### Identification and quantification of cholesterol and 7-ketocholesterol in egg yolks and cakes

The evaluation of cholesterol and 7-ketocholesterol (7-KC) was performed following the procedure reported by Innosa et al [19]. About 10 g of each cake were weighed into a 50 mL tube and 100 μL of stigmasterol was added as internal standard. The samples were saponified with 10 mL of ethanol, 500 μL of BHT 20% in methanol solution and 100 μL of KOH 50% water solution at 60°C in a water bath for 20 min. After cooling at room temperature, 2 mL of distilled water, 8 mL of petrolatum ether and 1 g of NaCl were added to each sample. Then, the samples were sonicated and centrifugated to separate into phase. The supernatant containing non-saponifiable lipids as cholesterol and cholesterol oxidation products were recovered into round bottom flasks. Other 2 mL of distilled water and 16 mL of petrolatum ether were added, and the phases were again allowed to separate. After recovering the supernatant, a third and last extraction was repeated on each sample. Then, the solvent was evaporated to dryness with a Strike-Rotating Evaporator (Steroglass s.r.l., Perugia, Italy) at 38°C and the non-saponifiable lipids were recovered with 1 mL of hexane and transferred into an 8-mL vial. At this point, the samples were evaporated for a second time with a nitrogen flow and derivatized with pyridine and BSTFA (N, O-bis (trimethylsilyl)-trifluoroacetamide) in 2:1 ratio. Then, samples were evaporated and recovered with 1 mL of hexane. The analyzes were conducted using a Focus GC (Thermo Scientific, USA) equipped with a FID and a Restek XTI-5 30 m, 0.32 mm ID, 0.25 μm as column. Split injection (20 mL/min split flow) was performed at 280°C. The oven temperature was held at 175°C for 1 min, increased to 230°C at 5°C/min and then to 300°C at 10°C/min and held for 5 min. Hydrogen was used as a carrier gas at a flow rate of 1 mL/min. Detector temperature was held at 280°C. Peak areas were quantified using ChromeCard software (Thermo Fisher Scientific, Waltham, MA, USA). The analytical method for the identification of cholesterol was calibrated in the range from 100 to 700 μg/mL (R^2^ = 0.9849) while the analytical method for the identification of 7-KC was calibrated in the range from 0.5 to 5 μg/mL (R^2^ = 0.9939). Each level of concentration was repeated 5 times and the limit of quantification was found as 0.5 μg/mL and the limit of detection was found as 0.15 μg/mL.

### Analysis of volatile profile in eggs and cakes

Extraction and analysis of volatile compounds (VOC) from egg yolks and cake samples was performed following the procedure previously reported by Ianni et al [20] with few modifications. The extraction was performed by solid-phase microextraction, and gas chromatography-mass spectrometry (GC-MS) analysis was performed with a GC (Clarus 580, Perkin Elmer, Waltham, MA, USA) coupled with a mass spectrometer (SQ8S, Perkin Elmer, USA). The GC was equipped with an Elite-5MS column (length×internal diameter: 30× 0.25 mm; film thickness: 0.25 μm; Perkin Elmer, USA). Four grams of previously chopped yolk or cake were mixed with 10 mL of saturated NaCl solution (360 g/L), then 10 μL of internal standard solution (4-meth-yl-2-heptanone; 10 mg/kg in ethanol) were added. The vials were sealed with a polytetrafluoroethylene-silicone septum (Supelco, Bellefonte, PA, USA) and stirred at 60°C; VOC were extracted from the headspace with a divinylbenzene-carboxen-polydimethylsiloxane solid-phase microextraction fiber (length: 1 cm; film thickness: 50/30 μm; Supelco, USA) with an exposition time of 50 min. After adsorption time, the extracted VOC were thermally desorbed into the GC injector splitless mode for 1 min at 250°C. The oven temperature was held at 50°C for 1 min, increased at a rate of 3°C/min up to 200°C and held for 1 min, and then increased from 200°C to 250°C at 15°C/min and held for 15 min. Helium was used as a carrier gas at a flow rate of 1 mL/min. The mass spectrometer operated in electronic impact ionization mode at 70 eV, and data were collected in full scan mode, with a scan time of 0.2 s over a mass range of 35 to 350. Source and interface temperature were held at 250°C. Volatile compounds were identified by comparison with mass spectra of a library database (NIST Mass Spectral library, Search Program version 2.0, National Institute of Standards and Technology, US Department of Commerce, Gaithersburg, MD, USA) and by comparing the eluting order with Kovats indices.

### Statistical analysis

All experiments were performed at least in triplicate and the results were reported as mean±standard deviation. The SigmaPlot 12.0 software (Systat software, Inc., Point Richmond, CA, USA) for Windows operating system was used to analyze the statistical significance of the differences between the averages for each group (analysis of variance). Student’s t-test was applied for the statistical evaluation of the differences observed in the analysis of diets (β-carotene, total flavonoids, AA and FA profile). Statistical significance is defined when p values are less than 0.05.

## RESULTS

### Characterization of extruded linseed and diets

The analysis on eggs and related food systems were preceded by the chemical characterization of both the EL used for dietary supplementation and the diets administered to animals during the 84 days of the trial (Table 1). Between the two dietary regimens, no differences were found in the amount of β-carotene, while a significant increase in total flavonoids was detected in the EG samples (16.02 vs 25.79 μg QUE/mL for CG and EG, respectively; p<0.01). Despite this evidence, no changes were found in the antioxidant potential that was similar in the feeds administered to the two EGs (p>0.05).

The analysis of FA profile highlighted, as expected, the high content of ALA (C18:3; 53.77%±3.52%) in the EL used as supplement for the EG diet; as a direct consequence of this finding, a higher presence of this compound was found in the experimental feed compared to the control diet (8.07% vs 4.04% in EG and CG feed samples, respectively; p<0.01). The other significant variations found in the diets administered during the trial concerned oleic acid (C18:0) and linoleic acid (LA) (C18:2). The first was greater in the EG samples (5.53%±0.47% vs 3.77%±0.29%; p<0.05), whereas the second was most represented in the CG samples (55.56%± 4.12% vs 46.90%±3.78%; p<0.05).

### Percentage of deposition and eggs physical characteristics

As reported in Table 2, the administration of the two diets showed no differences between the two groups neither for food and water intake nor for the average deposition percentage, which, at the end of the 84 days, was 85.2% in the CG and 86.5% in the EG. At the end of the trial, the average weight of whole eggs was greater in the group fed with linseed supplementation (58.05±1.94 g vs 63.37±2.14 g for CG and EG, respectively; p<0.05), while the egg components (yolk, albumen, and shell) showed an overlapping proportionality in the two groups (p>0.05).

The analysis of the egg yolks color (Table 3), through the CIELAB system, evidenced no differences between CG and EG in the lightness (L*). With regard to the chromatic coordinates (a*, b*, and c*), was significant the increase of the a* variable in EG yolks, which in fact tend to a reddish color tone, therefore darker than CG samples (2.58±0.22 vs 4.23±0.10 for CG and EG, respectively; p<0.05). Finally, the parameter indicating the tint (h) was greater in commercial eggs (p<0.05).

### β-Carotene, total flavonoids, and antioxidant activity in egg yolks and cakes

The linseed dietary intake had effects on the β-carotene content at the level of the yolks obtained from the collected eggs. Specifically, this compound was found to be more present in the EG samples (47.82±1.59 vs 32.48±1.38 μg/g in EG and CG, respectively; p<0.01). In the same samples a marked increase in total flavonoids was also detected (27.98±1.20 vs 23.54±1.91 μg QUE/mL in EG and CG, respectively; p<0.05) and, contrary to expectations, no changes were noted regarding the antioxidant potential (p>0.05).

In the cakes the same phenomenon as found in eggs was observed; the flavonoid content of EG increased (18.72±0.94 vs 16.82±0.87 μg QUE/mL in EG and CG, respectively; p< 0.05), while there was no variation in the antioxidant activity (p>0.05).

### Fatty acid profile and oxidative stability in egg yolks and cakes

The egg yolks obtained from both EGs showed no differences in the total lipid content, while interesting variations were evidenced by the FA profile analysis (Table 4). The linseed dietary intake induced a marked decrease of SFA, especially due to the reduction of palmitic acid (C16:0; p<0.05). There were no variations in MUFA and PUFA, although in this last case some differences between individual FAs were found. Specifically the EG samples were poorer in LA (C18:2; p< 0.01) but richer in ALA (C18:3; p<0.01); this result justified the considerable reduction of the ω-6/ω-3 ratio which reached 4.3:1 in EG samples against the 91.5:1 observed in the CG.

In the case of cakes the situation showed some differences compared to egg yolks. No variations were evidenced for SFA and, in the MUFA group, a marked increase of oleic acid (C18:1) was detected in EG samples (40.08%±0.83% vs 35.66%±1.75% in EG and CG, respectively; p<0.01). With regard to the PUFA the situation was similar to that observed in egg yolks: C18:2 was less represented in EG samples (p< 0.01), whereas, in the same samples, an increase in C18:3 was detected (p<0.05).

The evaluation of lipid oxidation in the egg yolks collected from both the EGs did not reveal any significant difference between the CG and the EG samples (0.69±0.09 vs 0.72±0.06 μg MDA/g for CG and EG, respectively; p>0.05).

With regard to cakes, the situation was quite different (Table 5). A general increase in the oxidation degree was observed as the duration of the heat treatment increased; however, the most interesting data concerned the direct comparison between the two EGs. At the level of raw doughs, a higher oxidation degree was observed in CG samples (1.55±0.13 vs 0.96±0.15 μg MDA/g for CG and EG, respectively; p<0.05). After 15 minutes of cooking the difference between the two groups disappeared, whereas, from the 30 minutes of cooking onwards, the EG samples showed a considerably higher oxidation than that characterizing the CG samples (p<0.01 and p<0.05 for 30 and 45 minutes, respectively).

### Cholesterol and 7-ketocholesterol in egg yolks and cakes

With regard to cholesterol (Table 6), the enrichment of laying hens diet with EL contributed to the production of eggs with a lower cholesterol amount with respect to the CG samples. That condition was confirmed in the cake doughs, in which cholesterol amounted on values that differed significantly between the two EGs (3.52±0.37 vs 2.73±0.28 mg/g for CG and EG, respectively; p<0.05).

The evaluation of cholesterol oxidation (Table 6) was performed through the identification of 7-KC, one of the most representative oxysterols. This analysis highlighted an interesting condition, in which the production of 7-KC seemed to be directly affected by the extent of the heat treatment. In the EG samples the 7-KC was detected only after longer heat treatment (45′), and it was interesting to note that its amount was still significantly lower than that identified in the CG samples in the same conditions (1.50±0.12 vs 0.72±0.07 μg/g for CG and EG, respectively; p<0.01).

### Volatile compounds

The analysis of the volatile profile in egg yolks and raw cake doughs did not lead to identifying any compound in the samples of both EGs.

The most interesting results were obtained from the evaluations carried out on cake samples obtained after 15, 30, and 45 minutes of cooking, in which a total of 21 compounds were identified (Table 7). The most represented family of compounds was that of aldehydes, in which was evidenced an increase of heptanal in the EG samples after 15 (p<0.05) and 30 (p<0.01) minutes of cooking, in the same samples hexanal increased after 30 (p<0.05) and 45 (p<0.01) minutes of cooking, while the nonanal showed a significant variation only after 45 minutes of cooking (p<0.01), reaching higher values in samples, also in this case, obtained after dietary supplementation with EL. Another group of compounds in which significant differences occurred is that of aromatic aldehydes. Of particular note was a greater presence of benzaldehyde in CG samples after 15 (p<0.05) and 30 (p<0.01) minutes of cooking, and the increase in concentration of ethyl vanillin in EG samples after 15 (p<0.05) and 30 (p<0.01) minutes of cooking, whereas no significant differences were evidenced between the two EGs after the longest cooking time interval. Finally, there was a significant reduction of ethyl benzene in EG samples after 30 minutes of cooking (p<0.01).

## DISCUSSION

The data reported in the present study represent the result of the analyzes carried out on samples obtained from three distinct samplings (28th, 56th, and 84th day) performed during the trial. The feeding strategy based on EL supplementation showed its effects on chemical-physical properties of eggs collected in the first sampling, and no change in the trend of the various parameters occurred in samples collected later in the trial. The ability of this feeding strategy to induce its effects in a fairly limited time is in full agreement with what was previously observed by Nain et al [21] who demonstrated that PUFA content in blood and eggs obtained from hens fed with a linseed supplementation reached a plateau in a very short time, even after 6 days of administration.

The dietary supplementation of laying hens with EL showed no differences between the two groups neither for food and water intake nor for the average deposition rate. The result concerning the deposition rate was slightly lower than the expected parameters, both in the CG and in the EG. Probably this was due to the trial being conducted during the winter season (December to March) in which particularly cold temperatures were recorded that could have affected the animals both during arrival and settling, and in the breeding phase [22]. Beyond this, the fact that no differences were recorded between the EGs is in total agreement with what was previously reported in other studies in which a dietary supplementation with flaxseed did not cause reductions in percentage of deposition or food intake [23,24]. In addition to this, the average weight of whole eggs was greater in the group that received the EL supplementation, while egg components in the two groups showed similar proportionality. This finding suggests an influence of EL in improving the production and commercial parameters, without modifying albumen and yolk percentage, according to other studies that integrated linseed in the hen’s diet in moderate to high percentage [25]. The analysis of physical properties also highlighted the role of the experimental diet in producing eggs with yolks characterized by a darker, reddish color. Generally, this result can be attributed to the presence of a greater quantity of carotenoids. In particular, the red color is usually associated with the presence of canthaxanthin, although β-carotene also seems to affect thisparameter. Effectively, β-carotene was more concentrated in the EG yolks, but as the analyzes on feeds did not show significant differences of this compound, it is plausible that β-carotene was assimilated from the feed with greatest efficiency by animals that received the EL supplementation. This process depends on many factors and absorption in the intestine is a major determinant of this process. In mammals, carotenoid absorption can be divided into four stages: digestion of the food matrix, formation of lipid-mixed micelles, uptake of carotenoids into the intestinal mucosal cells and delivery to the plasma via the lymph system [26].

The dietary supplementation with EL also modified the FA profile in eggs yolks and in the corresponding processed products; especially there was a marked decrease of SFA and LA, and an increase in ALA, whose percentage was almost 16 times higher in the EG egg yolks and more than 2 times higher in processed products. This data has interesting implications for consumers health. Indeed, the SFA, and especially the lauric, myristic and palmitic acids, appear predispose to the development of pathological conditions affecting the cardiovascular system, mainly due to the increase in plasma concentration of total cholesterol and low-density lipoprotein associated with cholesterol [27]. The high content of ALA and the reduction of LA represent an important finding because both FAs compete as substrate for the same enzyme, Δ6-desaturase, the first for the synthesis of anti-inflammatory and cardioprotective long chain FAs such as EPA and DHA, the second for the synthesis of arachidonic acid, precursor of leukotrienes and inflammatory prostaglandins [28]. Indeed the optimal ratio of long chain PUFAs ω-6/ω-3 to adequately perform their functions is ideally 1:1, but the diet in most developed countries drastically altered this relationship by unbalancing it in favor of the ω-6 for different reasons including the increased consumption of vegetable oils to limit atherosclerosis (corn, sunflower and peanuts, rich in ω-6 such as LA), the limited consumption of fish and ae lower presence of ω-3 in farmed fish due to lack of phytoplankton intake and the minimum amounts of ω-3 FAs in meat from domestic cattle [29]. Excessive amounts of omega-6 PUFA and a very high omega-6/omega-3 ratio, is reported to promote the pathogenesis of many diseases, including cardiovascular disease, cancer, and inflammatory and autoimmune diseases, whereas increased levels of omega-3 PUFA (a lower ω-6/ω-3 ratio), exert suppressive effects [30]. In this study, the dietary EL supplementation in laying hens led to the production of egg yolks in which was found a ω-6/ω-3 ratio equal to 4.3/1. As reported by De Lorgeril et al [31] an experimental diet providing a 4/1 ratio of LA/ALA led to a 70% decrease in total mortality of patients affected by cardiovascular disease.

No differences were found in lipid oxidation of egg yolks, while in cakes, obtained by cooking at 180°C at different time intervals, oxidation was observed, especially in the EG samples. This data undoubtedly indicates that the pro-oxidant factor is represented by the heat treatment and its influence on chemical stability of the analyzed samples. However, the fact that oxidation mainly attacks EG samples deserves special attention. Lipid oxidation generally occurs in matrices rich in PUFAs, which are more susceptible to the oxidative process. The EG samples do not have a greater amount of this subset of FAs with respect to the CG samples, but the marked increase in ALA, probably led to a variation in the relative composition of PUFAs, in favor of structures with a higher degree of unsaturation and therefore more susceptible to peroxidation. Evidence provided by Bandyopadhyay et al [32] showed that the oxidation rate is influenced by the PUFA composition; their study demonstrated that ALA was preferentially oxidized than LA in the liver of carnivorous catfish (*Heteropneustes fossilis*).

The finding of lipid oxidation is also useful to explain the data concerning the cholesterol oxidation in cakes, which is lower in the EG samples. As reported by Ansorena et al [33] and more recently by Innosa et al [19] who investigated the conditions of 7-KC formation in matrices of similar composition, the cholesterol oxidation appears to be secondary compared to that of PUFAs, so in these cases the cholesterol oxidation occurs in a less efficient way. In the present study this concept is amplified by the fact that in the EG samples a reduced amount of total cholesterol was identified compared to the CG samples, so the analyzed matrix offered less oxidizable substrate.

The analysis of the volatile profile of cakes showed interesting differences not only in the comparison between samples obtained from the EGs, but even in the presence of different cooking times. The most represented class of compounds is that of aldehydes at the level of which were found variations in hexanal, heptanal and nonanal. Hexanal is a compound typically derived from the oxidation of LA and in some circumstances it is also considered a reliable marker of oxidation [34]; in literature it is described as one of the main determinants of strong odors, sometimes unpleasant, perceived even in foods with a lower odor threshold. The fact that this compound tends to increase in EG samples only at 30 and 45 minutes of cooking, indicates that the oxidative process at the base of its synthesis is not sensitive to small heat treatments. Heptanal and nonanal which increased in EG samples, with their synthesis being generally associated with self-oxidation events affecting oleic acid [35]. As shown above, in the EG doughs, oleic acid is present in significantly higher concentrations and probably this evidence could justify the observed phenomenon. Another group of compounds with significant differences is the aromatic aldehydes. Of particular note is the increase in ethyl vanillin in the EG samples. This finding is not entirely surprising, because vanillin, to which are associated pleasant and relatively intense aromatic notes, is the phenolic compound most represented in flaxseeds [36]. However, in this case it must be emphasized that the difference observed after 15 and 30 minutes of cooking cannot be found after 45 minutes, reflecting the fact that the intense heat treatment may have degraded the compound, presumably worsening the aromatic properties of the processed product. Finally no varitations were identified for alcohols, ketones, and carboxylic acids, whose contribution to the definition of the aromatic profile, therefore, needs further investigations.

## Figures and Tables

**Table 1:** Chemical characterization of extruded linseed and diets administered to laying hens of control and experimental group

Items	Diet		Extruded linseed

	CG1)	EG1)	
Chemical composition (%)			
Dry matter	89.33	88.91	-
Crude protein2)	19.07	18.52	27.33
Ether extract2)	5.77	5.95	37.84
Fiber2)	3.24	3.44	9.43
Ash2)	15.01	15.21	3.85
Antioxidants			
β-carotene (μg/g)	27.73±1.43	28.38±1.56	41±2.31
Total flavonoids (μg QUE/mL)	16.02^A^±0.82	25.79^B^±1.49	35.58±2.64
AA (TEAC μmol/mL)	31.95±2.13	31.78±2.43	45.68±3.15
Fatty acid profile (%)			
C16:0	11.78±0.95	13.26±0.96	6.24±0.48
C18:0	3.77^a^±0.29	5.53^b^±0.47	4.68±0.34
C18:1	24.85±2.21	26.26±2.08	19.72±1.51
C18:2	55.56^A^±4.12	46.90^B^±3.78	15.59±1.33
C18:3	4.04^A^±0.32	8.07^B^±0.67	53.77±3.52

Data are reported as mean±standard deviation.

DM, dry matter; QUE, quercetin; AA, antioxidant activity; TEAC, Trolox equivalent antioxidant capacity.

1) CG, control group; EG, experimental group.

2) On a dry matter basis.

Values in the same row followed by different letters indicate significant difference between diets (^a,b^ p<0.05; ^A,B^ p<0.01).

**Table 2:** Productive parameters evaluated during the experimental period on the group of animals fed with a standard diet and those fed a supplementation of extruded linseed

Treatment period (d)	Hen weight (kg)		Feed consumption (g/head/d)		Deposition index (%)		Egg weight (g)	
			
	CG1)	EG1)	CG	EG	CG	EG	CG	EG
0 to 28	1.68–1.79	1.70–1.78	109–114	110–116	84.2	84.7	55.78^a^±1.69	59.97^b^±1.82
29 to 56	1.83–1.92	1.80–1.93	115–119	116–120	85.6	86.0	56.93^a^±1.73	62.43^b^±1.91
57 to 84	1.89–1.99	1.94–2.00	120–124	120–123	85.2	86.5	58.05^a^±1.94	63.37^b^±2.14

1) CG, control group; EG, experimental group.

a,b For each parameter, different letters in the same row indicate significant differences between experimental groups (p<0.05).

**Table 3:** Coloration of egg yolks obtained from control and experimental group

Items	CG1)	EC1)	p-value
L*	17.86±0.47	17.73±0.56	n.s.
a*	2.58±0.22	4.23±0.11	**
b*	23.41±0.58	24.05±0.77	n.s.
c*	23.55±0.58	24.42±0.77	n.s.
h	83.66±0.46	79.96±0.31	*

Data are reported as mean±standard deviation for ten replicates.

1) CG, control group; EG, experimental group.

L*, lightness (0 = black, 100 = white); a*, redness (–100 = green, 100 = red); b*, yellowness (–100 = blue, 100 = yellow); C*, chroma or saturation; h, hue angle.

* p<0.05;

** p<0.01; n.s., not significant.

**Table 4:** Total lipids and fatty acid profile of egg yolks and baked products obtained from control and experimental group

Items	Egg yolk			Baked product		
	
	CG1)	EG1)	p-value	CG	EG	p-value
Total lipids2)	25.71±2.18	22.35±1.83	n.s.	8.91±0.80	9.15±0.37	n.s.
C14:0	0.13±0.02	0.26±0.02	n.s.	n.d.	n.d.	
C16:0	26.73±2.17	21.54±1.84	*	24.68±0.54	23.73±0.25	n.s.
C18:0	7.56±0.49	8.26±0.65	n.s.	9.53±0.51	9.11±0.56	n.s.
SFA	34.42±1.75	30.06±1.07	*	34.21±1.05	32.84±0.96	n.s.
C16:1	2.30±0.41	3.25±0.38	n.s.	1.97±0.12	2.16±0.16	n.s.
C18:1	42.93±2.77	46.53±3.02	n.s.	35.66±1.75	40.08±0.83	*
MUFA	45.23±2.04	49.78±2.19	n.s.	37.63±2.08	42.24±2.23	n.s.
C18:2	18.74±1.77	14.27±1.15	**	24.97±1.03	20.52±0.63	**
C18:3	0.22±0.03	3.57±0.32	**	1.27±0.07	3.08±0.30	*
C20:4	1.39±0.17	1.08±0.13	n.s.	n.d.	n.d.	
PUFA	20.35±1.96	18.92±1.61	n.s.	26.24±2.12	23.60±1.81	n.s.

Data are reported as mean±standard deviation for ten replicates.

SFA, saturated fatty acid; MUFA, monounsaturated fatty acid; PUFA, polyunsaturated fatty acid; n.s., not significant; n.d., non detectable.

1) CG, control group; EG, experimental group.

2) Values are reported on a dry matter basis.

Means are different at p<0.05 (*) and at p<0.01 (**).

**Table 5:** Lipid oxidation in cakes baked with different cooking times (15, 30, and 45 minutes) and by using egg yolks from control and experimental group

Items		CG1)	EG1)
Cake dough		1.55^a^,^A^±0.13	0.96^b^,^A^±0.15
Cooking time	15 min	4.55^B^±0.35	4.64^B^±0.31
	30 min	5.01^a^,^B^±0.37	8.50^b^,^C^±0.64
	45 min	6.18^a^,^C^±0.43	7.93^b^,^C^±0.55

Data are reported as mean (μg malondialdehyde/g)±standard deviation for ten replicates.

1) CG, control group; EG, experimental group.

a,b Different lowercase letters in the same row indicate significant differences between the two groups reported as mean (μg malondialdehyde/g)±standard deviation (p<0.05).

A–C Different capital letters in the same column (group) indicate significant differences between cooking times (p<0.01).

**Table 6:** Cholesterol and 7-ketocholesterol in egg yolks and cakes subjected to different cooking times (15, 30, and 45 minutes)

Items		CG1)	EG1)	p-value
Cholesterol (mg/g)				
Egg yolk		17.06±2.08	13.40±1.50	*
Cake dough		3.52±0.37	2.73±0.28	*
7-Ketocholesterol (μg/g)				
Cake dough		n.d.	n.d.	
Cooking time	15 min	0.65^a^±0.07	n.d.	
	30 min	0.70^a^±0.08	n.d.	
	45 min	1.50^b^±0.12	0.72±0.07	**

Data are expressed as mean±standard deviation for ten replicates.

1) CG, control group; EG, experimental group.

* p<0.05;

** p<0.01;

n.d., not detectable.

a,b Different letters in the same column (group) indicate significant differences between cooking times.

**Table 7:** Volatile compounds detected in cupcakes subjected to different cooking times (15, 30, and 45 min) and prepared by using eggs obtained from control and experimental group

VOC	15 min			30 min			45 min		
		
	CG	EG	p-value	CG	EG	p-value	CG	EG	p-value
Butanal, 2-methyl	4.35±0.35	4.71±0.37	n.s.	3.20±0.26	3.54±0.25	n.s.	4.71±0.41	4.55±0.39	n.s.
Butanal, 3-methyl-	7.43±0.63	8.85±0.84	n.s.	5.15±0.42	6.03±0.54	n.s.	8.05±0.72	7.05±0.67	n.s.
Decanal	0.27±0.03	0.23±0.02	n.s.	0.17±0.02	0.11±0.02	n.s.	0.19±0.02	0.16±0.02	n.s.
Furfural	36.48±3.43	39.91±2.07	n.s.	50.53±3.98	49.21±3.22	n.s.	35.58±3.66	39.52±1.25	n.s.
Heptanal	0.49±0.05	0.66±0.07	*	0.48±0.05	1.37±0.11	**	0.17±0.02	0.20±0.02	n.s.
Hexanal	4.32±0.38	4.11±0.39	n.s.	1.79±0.16	2.52±0.29	*	1.21±0.11	1.96±0.17	**
Nonanal	2.80±0.26	2.24±0.32	n.s.	1.68±0.15	1.73±0.14	n.s.	0.69±0.07	0.98±0.09	**
Propanal, 2-methyl	0.87±0.09	1.09±0.10	n.s.	0.94±0.09	0.99±0.10	n.s.	1.43±0.13	1.47±0.15	n.s.
1-undecanol	0.42±0.04	0.42±0.05	n.s.	0.32±0.03	0.27±0.03	n.s.	0.33±0.03	0.28±0.03	n.s.
Maltol	n.d.	n.d.		0.37±0.04	0.43±0.04	n.s.	0.82±0.08	0.69±0.07	n.s.
2-Furanmethanol	9.59±0.87	9.83±0.88	n.s.	13.46±1.07	13.29±1.13	n.s.	15.72±1.23	15.53±1.15	n.s.
2-Furanmethanol, 5-methyl	0.24±0.03	0.18±0.02	n.s.	0.55±0.05	0.44±0.04	n.s.	0.43±0.04	0.40±0.04	n.s.
Benzaldehyde	3.19±0.25	2.59±0.24	*	0.84±0.08	0.35±0.04	**	6.83±0.63	7.61±0.56	n.s.
Benzeneacetaldehyde	1.76±0.14	1.59±0.17	n.s.	2.54±0.19	2.75±0.24	n.s.	3.34±0.28	3.85±0.35	n.s.
Ethyl vanillin	1.19±0.11	1.54±0.13	*	0.36±0.04	0.63±0.06	**	0.36±0.04	0.45±0.05	n.s.
Ethylbenzene	7.96±0.69	6.74±0.64	n.s.	3.34±0.27	2.43±0.17	**	3.72±0.34	4.54±0.39	n.s.
P-xylene	5.41±0.45	4.62±0.41	n.s.	2.14±0.18	1.92±0.17	n.s.	2.76±0.24	2.83±0.25	n.s.
Ethanone, 1-(2-furanyl)	n.d.	0.40±0.04		0.99±0.10	1.00±0.09	n.s.	0.56±0.07	0.54±0.05	n.s.
2,3 Pentanedione	0.23±0.03	0.27±0.03	n.s.	0.28±0.03	0.30±0.03	n.s.	0.50±0.05	0.46±0.05	n.s.
Pyranone	n.d.	n.d.		0.25±0.03	0.18±0.02	n.s.	0.30±0.03	0.28±0.03	n.s.
Pentanoic acid	0.44±0.04	0.53±0.05	n.s.	0.64±0.06	0.42±0.05	n.s.	0.31±0.04	0.25±0.02	n.s.

Data are expressed as mean (%)±standard deviation for five replicates.

VOC, volatile compounds; CG, control group; EC, experimental group.

* p<0.05;

** p<0.01;

n.s., not significant; n.d., non detectable.
